# Impact of Placental *Plasmodium falciparum* Malaria on the Profile of Some Oxidative Stress Biomarkers in Women Living in Yaoundé, Cameroon

**DOI:** 10.1371/journal.pone.0134633

**Published:** 2015-08-12

**Authors:** Rosette Megnekou, Jean Claude Djontu, Jude Daiga Bigoga, Fabrice Mbah Medou, Sandrine Tenou, Abel Lissom

**Affiliations:** 1 Department of Animal Biology and Physiology of the Faculty of Sciences, University of Yaounde I, Yaoundé, Cameroon; 2 The Biotechnology Center, University of Yaounde I, Yaounde, Cameroon; 3 Department of Biochemistry of the Faculty of Science, University of Yaounde I, Yaoundé, Cameroon; Institut de Recherche pour le Développement, FRANCE

## Abstract

**Background:**

Impact of the pathophysiology of *Plasmodium falciparum* placental malaria (PM) on the profile of some oxidative stress biomarkers and their relationship with poor pregnancy outcomes in women remain unknown.

**Methods:**

Between 2013 and 2014, peripheral blood and placenta tissue from 120 Cameroonian women at delivery were assessed for maternal haemoglobin and, parasitaemia respectively. Parasite accumulation in the placenta was investigated histologically. The levels of oxidative stress biomarkers Malondialdehyde (MDA), Nitric Oxide (NO), Superoxide dismutase (SOD), Catalase (CAT) and Gluthatione (GSH) in the supernatant of teased placenta tissues were determined by Colorimetric enzymatic assays.

**Results:**

Parasitaemia was inversely related to haemoglobin levels and birth weight (P <0.001 and 0.012, respectively). The level of lipid peroxide product (MDA) was significantly higher in the malaria infected (P = 0.0047) and anaemic (P = 0.024) women compared to their non-infected and non-anaemic counterparts, respectively. A similar trend was observed with SOD levels, though not significant. The levels of MDA also correlated positively with parasitaemia (P = 0.0024) but negatively with haemoglobin levels (P = 0.002). There was no association between parasitaemia, haemoglobin level and the other oxidative stress biomarkers. From histological studies, levels of MDA associated positively and significantly with placenta malaria infection and the presence of malaria pigments. The levels of SOD, NO and CAT increased with decreasing leukocyte accumulation in the intervillous space. Baby birth weight increased significantly with SOD and CAT levels, but decreased with levels of GSH.

**Conclusions:**

Placental *P*. *falciparum* infection may cause oxidative stress of the placenta tissue with MDA as a potential biomarker of PM, which alongside GSH could lead to poor pregnancy outcomes (anaemia and low birth weight). This finding contributes to the understanding of the pathophysiology of *P*. *falciparum* placental malaria in women.

## Introduction

*Plasmodium falciparum* Placental malaria (PM) is a major cause of poor pregnancy outcomes in mothers (maternal anaemia and mortality) and their offspring (low birth weight, intra-uterine growth retardation and preterm delivery) [1]. In most cases, it is linked to placental tissue inflammation. Indeed, PM can be manifested as an acute condition with little or no inflammation, or as a chronic disorder, sometimes with heavy inflammation and deposition of parasite pigment [2]. The susceptibility of women to PM is attributed to increased parasite sequestration in the placenta, mediated by Chondroitin Sulfate A (CSA) binding to the syncytiotrophoblasts [3]. It can also be due to pregnancy-associated suppression of inflammatory responses caused by leukocyte infiltration in the intervillous space and the modulation of some biomarkers such as cytokines, chemokines and hormones in the placenta [4, 5, 6, 7]. Also, oxidative stress has been implicated in several placental disorders and pregnancy pathologies [8]. In fact, in normal cells, there is an appropriate pro-oxidant/anti-oxidant balance. Shifting of this balance towards the pro-oxidant side results in oxidative stress, manifested by elevated levels of free radicals and increase cell membrane lipid peroxidation (malondialdehyde –MDA). It is the lipid peroxide product whose level can be used as oxidative stress index), which is responsible for cell damage. Antioxidant systems which counteract this damage includes enzymes like superoxide dismutase (SOD), catalase (CAT), glutathione peroxidase (GSH); small molecules like Nitric oxide (NO), ascorbic acid, uric acid, β carotene, α tocopherol, reduced glutathione [9]. Although some information exists on the pathophysiological mechanisms implicated in the genesis of poor pregnancy outcomes associated to PM, there is none on placental malaria and oxidative stress biomarkers and their link to pregnancy outcomes. However, Tiyong Ifoue *et al*. [10] reported an imbalance between oxidants and antioxidants with regards to malaria in women during pregnancy.

Studies of the effects of malaria on oxidative stress related to placenta would contribute to a better understanding of the pathophysiology of PM and the identification of potentially new biomarkers for placental malaria. Studies have shown that antenatal diagnosis of PM by Giemsa-stained blood cannot capture all PM cases [11]. Nevertheless, this study reported only on parasitaemia, not on inflammation. Other studies have reported an association between plasma urokinase receptor levels and low birth weight in maternal malaria as well as positive correlation between plasma levels of TNF receptors 1 and 2 and *P*. *falciparum* parasitaemia [12, 13]. This suggests that host biomarkers may be useful in discriminating women likely to experience poor pregnancy outcomes. In this way, placental biomarkers of oxidative stress may be of particular value; since this condition is related to inflammatory response. Furthermore, MDA produced in the organism’s tissue as lipid peroxide product can be detected in peripheral plasma [14]. This study therefore aims to determine effect of PM on the profile of some oxidative stress biomarkers. The levels of MDA, GSH, CAT, SOD and NO were measured in teased placenta tissue supernatants of malaria infected and non-infected women at delivery.

## Materials and Methods

### Ethical consideration

Participation in the study was voluntary with written Informed Consent from each participant prior to recruitment. For women considered to be minors, written informed assent was obtained from their guardians. The National Ethics Committee of Cameroon approved the study (Ethical *Clearance* 2013/02/N^°^ 029/L/CNERSH/SP). Administrative Authorizations were obtained from the Ministry of Public Health of Cameroon (N^°^ D30-392 AAR/MINSANTE/SG/DROS/CRC/CEA1) and from the health centre. The study was performed in strict respect of the guidelines for human clinical research and as recommended by the Ministry of Public Health. Data was treated in the strict respect of anonymity.

### Study area and population

This study was cross sectional and took place between May 2013 and June 2014 at the Marie Reine Health Center in Etoudi, Yaounde, and southern forested Cameroon. Details of the study area and population as well as the sample collection process have been described elsewhere [6]. Briefly, all women attending the Health Center for delivery and who were willing to participate in the study were consecutively enrolled. After explaining the research project and obtaining a signed informed consent, information on the mother’s health, estimated length of pregnancy, parity, age, use of antimalarial drugs, HIV status, birth outcome and baby weight were recorded in a structured questionnaire. Immediately following delivery, peripheral blood samples (4ml) from women (n = 120) aged 16 to 39 years were aseptically collected into EDTA tubes and a portion used to prepare smears for malaria microscopy. Placental tissues were also collected for histology, for preparation of impression smears for malaria diagnosis and supernatants from teased portion of the tissue for the oxidative stress biomarker analysis. The characteristics of the study participants are summarized in Table 1.

**Table 1:** Characteristics of the study population.

Parameter	All women (N = 120)	Malaria infected (N = 18)	Malaria non-infected (N = 102)	P *
Age, median(range), years	26 (16–39)	25 (16–37)	27 (18–39)	0.32
Primipara	34 (28.3%)	06 (17.7%)	28 (82.3%)	
Secondipara	29 (24.2%)	06 (20.7%)	23 (79.3%)	
Multipara	57 (47.5%)	06 (10.5%)	51 (89.5%)	
Hb levels (g/dL)	12.4 (7.5–16)	10.4 (7.5–13.6)	12.8 (9.9–16)	<0.001
Anaemia	17 (14.2)	11 (64.7)	06 (35.3)	
Baby birth weight (g)	3400 (2100–4850)	3200 (2100–3700)	3500 (2200–4850)	0.017
Placental malaria by histology	16 (13.3%)	14 (77.8%)	02(2%)	<0.001
Leukocyte accumulation	22 (18.3%)	04 (22.2%)	18 (17.64%)	0.4
Malaria pigments	15 (12.5%)	12 (66.7%)	03 (2.9%)	<0.001

* The statistical significance between infected and non-infected women, using Mann- Whitney Rank Sum Test or Fisher exact test. Values in the parentheses represent either percentage or range where applicable.

### Determination of parasitaemia and haemoglobin levels

The impression smears (from placenta tissue) were stained using Giemsa and microscopically examined for the presence of *P*. *falciparum*. If parasites were not detected after examining 200 high power microscopic fields, the woman was considered non- infected. However, when parasites were detected, the woman was considered malaria infected and parasitaemia estimated by counting the number of infected erythrocytes per 200 RBCs and expressed in percentage. The smear was also used to determine the parasite species. Parasitaemia was further confirmed using malaria rapid diagnostic test (SD Bioline malaria antigen Pf/pan, Standard Diagnostics Inc, Kyonggi-do, Korea), a rapid diagnostic test for the detection of *P*. *falciparum*. Haemoglobin levels were determined using Coulter counter (URIT-3300, Europe) and a woman was anaemic if Hb < 11 g/dl, according to the manufacturer recommendation.

### Placental tissue examination by histology assay

The placental tissue was fixed by immersing in 10% buffered formalin. This was, then transferred into 70% ethanol, dehydrated, embedded in paraffin and 4μm section cut using a microtome. Sections were stained with haematoxylin and eosin, and slides examined by light microscopy. Haemozoin pigments were examined under polarized light to increase their visibility and minimize false positivity by formalin crystals. The presence of parasites, malarial pigments and leukocyte accumulation in the intervillous spaces was noted. Placental pathology was classified as either: i) no infection (no parasites or pigments found); ii) infected (presence of parasites); iii) malaria pigments (presence of either only malaria pigments or with malaria parasites); and iv) accumulation of leukocytes into the intervillous space of the placenta tissue.

### Measurement of oxidative stress biomarkers

A piece of placenta tissue (20% w/v) was weighed and teased in a mortar on ice-tray with Mc Even’s solution. Thereafter, the homogenate (from teased tissue) was centrifuged at 1200 rpm for 15mn at 4°C. The supernatant was collected and aliquoted in several tubes (to avoid freeze and thaw) and stored at -20°C for oxidative stress biomarker measurements. The supernatant levels of MDA, NO, SOD, CAT and GSH were determined by Colorimetric enzymatic methods on a spectrophotometer (Genesis, Suisse). Briefly, **MDA** was measured using thio-barbituric acid (TBA, 0.6%), Trichloro-acetic (TCA, 20%) and Tris-HCL buffers (HCl = 50mM and KCl = 150mM), in distilled water. The mixture was heated at 90°C for 10min, allowed to cool and centrifuged at 3000 rpm for 15min. The OD was read at 530 nm. [15]. **GSH** was measured using phosphate buffer (Na_2_HPO_4_, H_2_O and NaH_2_PO_4_, H_2_O, 0.1M) and 2, 2-dithio-5, 5-dinitrobenzoic acid (DTNB,). The mixture was incubated for 1h at 4°C and then centrifuged at 1200 rpm for 15min. The OD was read at 412 nm [16]. **Catalase** was measured using H_2_O_2_ phosphate buffer (0.1M), a mixture of potassium dichromate/glacial acetic acid (5% v/v) and hydrogen peroxide (50mM). The OD was read at 570 nm [17]. The level of **NO** was estimated as total nitrite (NO_2_) via Griess reaction in accordance with Marletta *et al* [18]. **SOD** was measured using carbonate buffer (Na_2_CO_3_, 10H_2_O and NaHCO_3_; 0.05M) and 0.3mM adrenaline solution. The mixture was homogenised and the OD read at 480nm [19].

### Statistical analyses

The GraphPad Prism 5.03 software was used for the statistical analyses. Results were reported as medians with 95% confidence intervals. Mann-Witney rank sum test was used to evaluate inter-group differences. Spearman rank order coefficient (r_s_) was used to evaluate parameter association. *P* values ≤ 0.05 were considered statistically significant.

## Results

### Study population

The baseline characteristics of the study population are summarized in Table 1. The prevalence of malaria diagnosed by microscopy was 13.3% (n = 16), 9.2% (n = 11) and 15.0% (n = 18) in peripheral blood, placental blood, and impression smear from the placenta tissue respectively. The latter was chosen as the prevalence of malaria at delivery for further analyses as focus is on placental malaria. The study confirms that primipara (17.7%) and secundipara (20.7%) women were more likely to be infected by *P*. *falciparum* compared to multipara (10.5%). Haemoglobin levels were lower in the infected compared to non-infected women (P <0.001) (Table 1). Parasitaemia increased significantly with decreasing haemoglobin levels and baby birth weight [(r_s_ = -0.46 and -0.23), P < 0.001 and 0.012), respectively](Table 2). Although a similar pattern was observed for the mother’s age and parity, this was not significant (r_s_ = -0.11, P = 0.23 for both) (Table 2). Median birth weight from malaria infected women were lower than in the non-infected (P = 0.017) (Table 1). These results depict the effects of *P*. *falciparum* infection on the prevalence of anaemia and confirm that babies born from infected mothers are more likely to have low birth weight compared to those non-infected. Histology results showed that 77.8% of infected women had active infection compared to 2% in women with negative impression smear (P <0.001). A similar trend was observed for the presence of malaria pigments in placental tissue (p <0.001), but different for the leukocyte accumulation in the intervillous space (p >0.05) (Table 1).

**Table 2:** Correlation between parasitaemia and haemoglobin levels, parity, mother’s age and baby birth weight.

Spearman rank order correlation [magnitude, 95% confidence intervals](N = 120)		
Parameter	r_s_	P-value
Mother’s age	-0.10[-0.29 to 0.08]	0.25
Parity	-0.11[-0.29 to -0.07]	0.23
Haemoglobin levels	-0.46[-0.60 to -0.30]	<0.0001
Baby birth weight	-0.23[-0. 40 to -0.05]	0.012

### MDA oxidative stress biomarker levels correlate positively with *P*. *falciparum* infections, but inversely with haemoglobin levels

**Fig 1:**
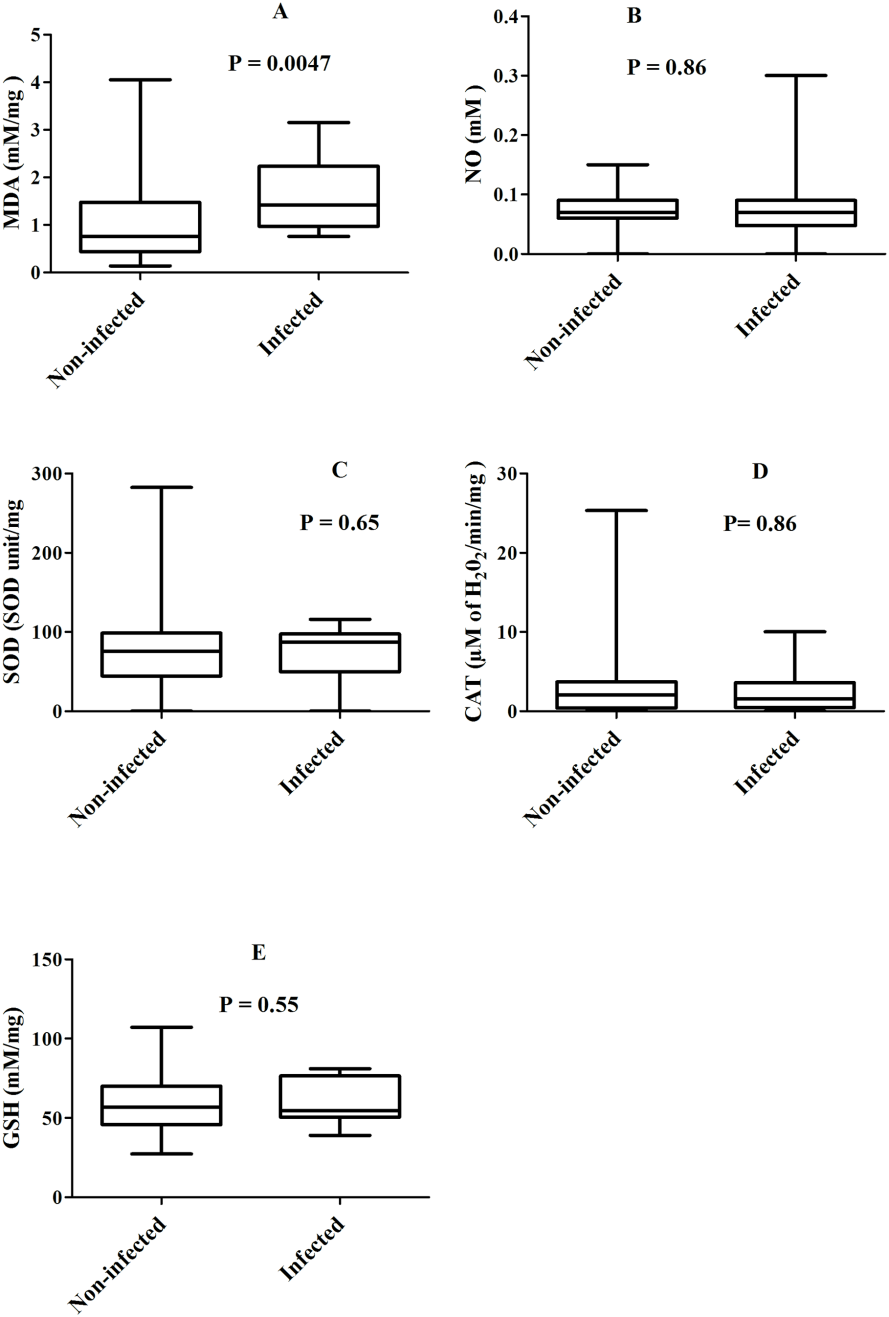
Levels of oxidative stress biomarkers between malaria infected and non-infected women. Box represents median with 25 and 75 percentiles.

Following the observed association between parasitaemia and haemoglobin levels (r_s_ = -0.46, P <0.0001), the relationship between oxidative stress biomarker levels with either parasitaemia or haemoglobin was determined in women at delivery. Thus, with regards to malaria infection, MDA levels were significantly higher in the malaria infected than the malaria non-infected women [median: 1.42 vs 0.76 mM/mg, P = 0.0047 (Fig 1A)], 95% confidence intervals: [1.24–1.94] vs [0.95–1.30]. A similar but not significant trend was observed with SOD levels [median 86.91 vs 75.61 SOD unit/mg, P > 0.5 (Fig 1C)], 95% confidence intervals: [61.74–92.10] vs [68.41–84.72]. On the contrary, there was no significant difference between malaria infected and non-infected women by considering the median levels of the other oxidative stress markers tested [median: NO = 0.07 vs 0.07 mM, CAT = 1.59 vs 2.10 μM of H_2_O_2_/min/mg and GSH = 54.78 vs 57.00 mM/mg; P > 0.5 for all (Fig 1B, 1D and 1E)], 95% confidence intervals: [0.05–0.11] vs [0.07–0.08] for NO and [52.00–68.87] vs [55.00–61.91] for GSH. Moreover, spearman rank order analysis shows that parasitaemia correlated positively with increased levels of MDA [(r_s_ = 0.26; P = 0.0024)], 95% confidence interval: [0.08–0.42]. No association was found between parasitaemia and the other oxidative stress markers tested.

**Fig 2:**
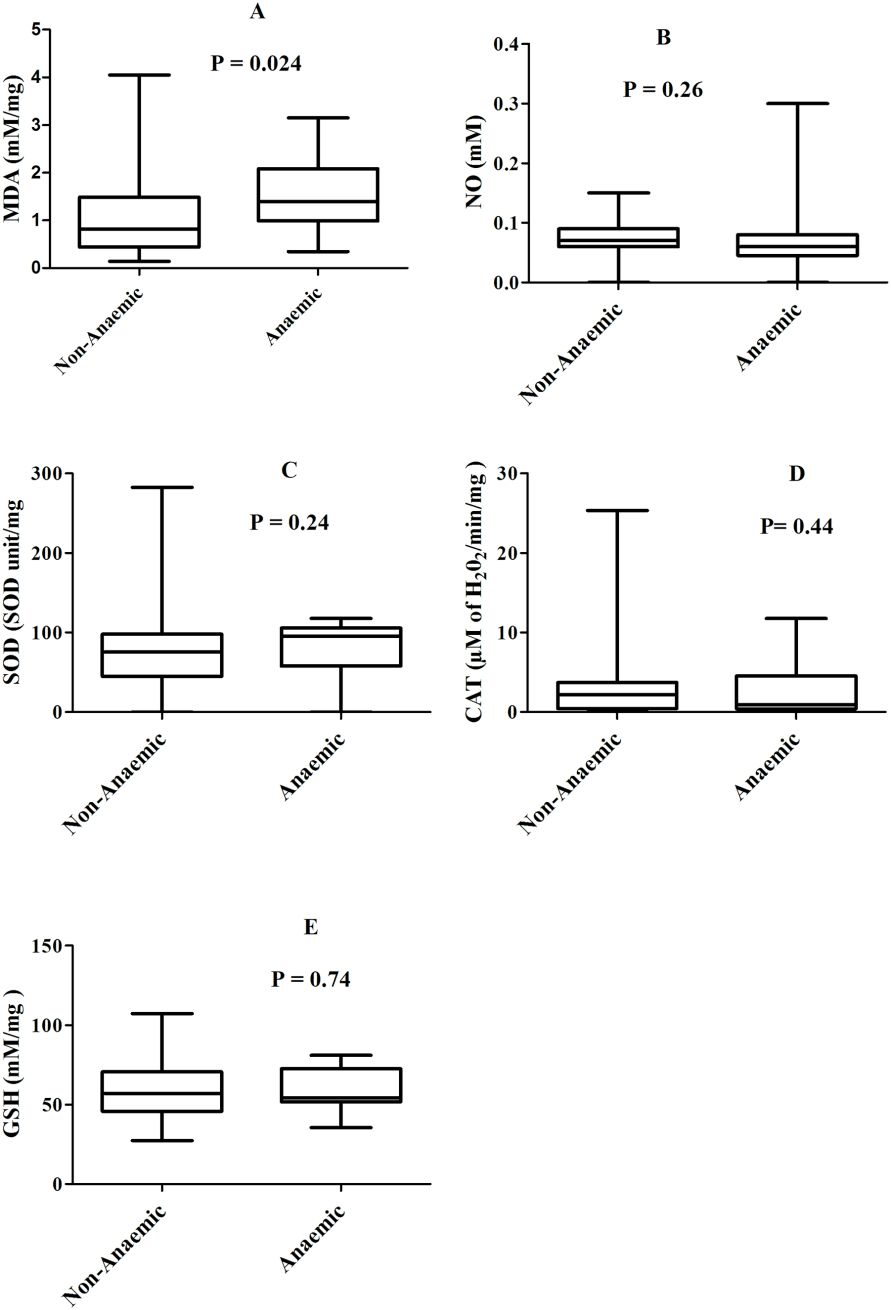
Levels of oxidative stress biomarkers between anaemic and non-anaemic women. Box represents median with 25 and 75 percentiles.

In comparison with the haemoglobin levels, MDA levels were higher in the anaemic than the non anaemic women [median: 1.40 *vs* 0.82 mM/mg, P = 0.024] (Fig 2A), 95% confidence intervals: [1.15–1.94] vs [0.97–1.32]. Although a similar trend was observed with SOD levels [median 95.33 vs 75.42 SOD unit/mg, P = 0.24 (Fig 2C)], 95% confidence intervals: [63.43–98.67] vs [67.89–83.88], this was not significant. The difference between anaemic and non anaemic women for the median levels of other oxidative stress biomarkers was not significant [median: NO = 0.06 vs 0.07 mM, CAT = 0.91 vs 2.22 μM of H_2_O_2_/min/mg and GSH = 54.26 vs 57.13 mM/mg; P > 0.2 for all (Fig 2B, 2D and 2E)], 95% confidence intervals: [0.04–0.10] vs [0.07–0.08] for NO and [51.47–66.74] vs [55.26–62.12] for GSH. Haemoglobin levels correlated negatively with levels of MDA (r_s_ = -0.28; P = 0.002) and positively with levels of NO (r_s =_ 0.18; P = 0.06), 95% confidence intervals: [-0.44–-0.10] for NO and [-0.01–0.35] for GSH. These results are suggestive of an association between placental *P*. *falciparum* infection and oxidative stress, which might contribute to the development of maternal anaemia.

### Association between plasma levels of oxidative stress biomarkers and other parameters (parity, mother’s age and baby birth weight)

This study showed that parasitaemia correlated significantly but inversely to haemoglobin levels and baby birth weight (P <0.0001 and 0.012, respectively). Although a similar trend was observed with parity and mother’s age, it was however not significant. Furthermore, correlation between parasitaemia or haemoglobin levels and some oxidative stress biomarkers were found. Therefore, the relationship with other parameters such as parity, mother’s age and the baby birth weight was determined using Spearman rank order correlation test. While levels of SOD and CAT correlated positively with baby birth weight [(r_s_ = 0.20; P = 0.03 for SOD) and [(r_s_ = 0.17; P = 0.06 for CAT), 95% confidence intervals: [0.014–0.37] for SOD and [-0.02–0.34] for CAT, those of GSH correlated negatively (r_s_ = -0.19; P = 0.04), 95% confidence interval: [-0.36–0.00]. A negative but not statistically significant correlation was observed for MDA and NO with baby birth weight, [(r_s_ = -0.13; P = 0.15 and r_s_ = -0.12; P = 0.19, respectively)].

Likewise, negative but not significant associations were found between parity and GSH, mother’s age and GSH as well as mother’s age and SOD (r_s_ = -0.15 to– 0.10; P = 0.11 to 0.26). These results suggest that high levels of the lipid peroxide product (MDA) and pro-oxidant nitric oxide and anti-oxidant, the glutathione in the placenta tissue might contribute to the impairment of foetal development while catalase might enhance foetal growth.

### Elevated levels of MDA associated positively with placenta malaria infection and the presence of malaria pigment while that of NO and SOD instead associated negatively with leukocyte accumulation in the placenta tissue

**Fig 3:**
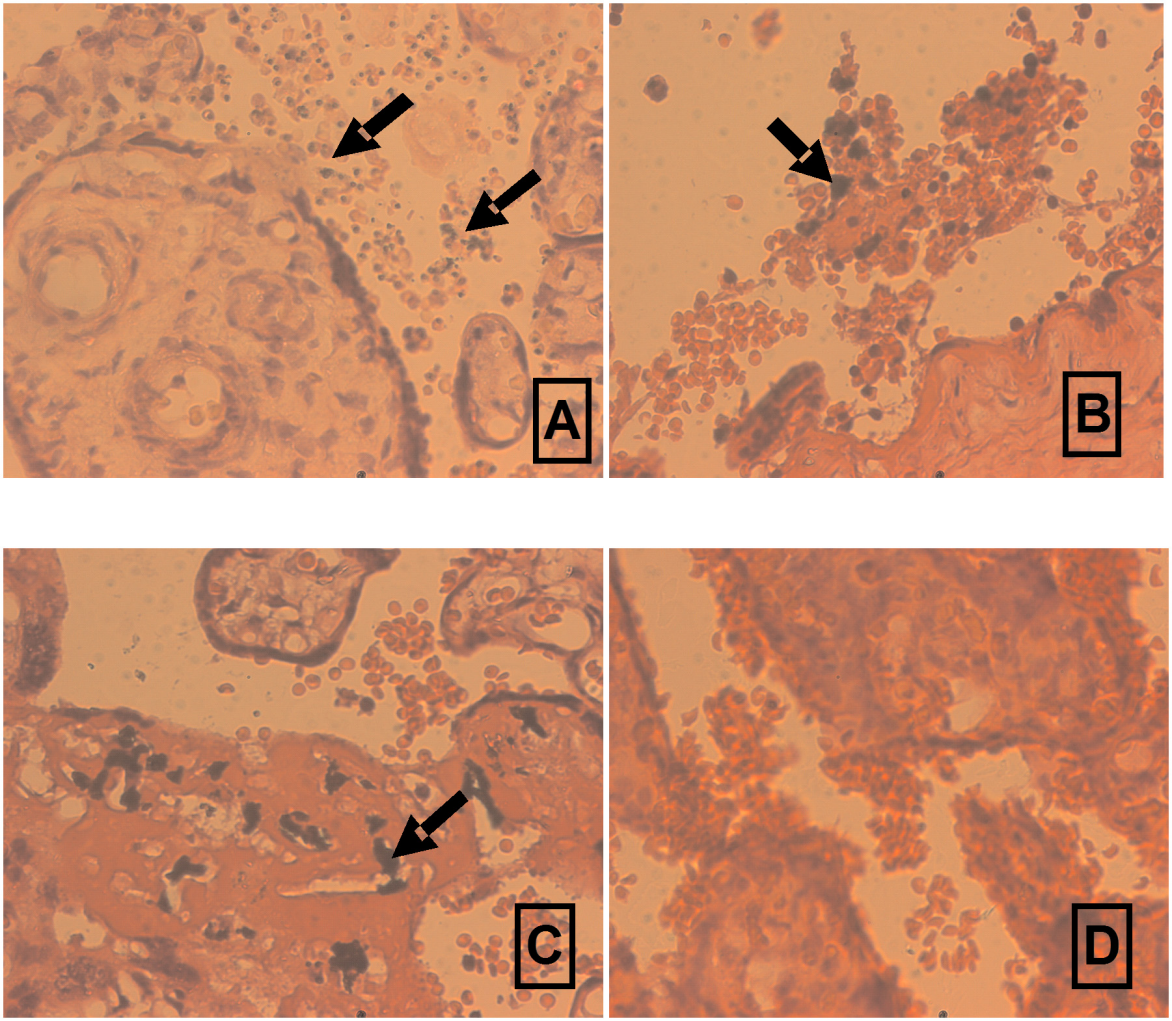
Different placental pathologies: *P*. *falciparum* parasitaemia (A and B), Leukocyte accumulation into the intervillous space (B), malaria pigments in fibrinoid (C), absence of none of the above mentioned pathology in women at delivery (D). Arrow indicates infected red blood cells (A), leukocyte accumulation into the intervillous space (B) and malarial pigments (C).

In order to determine whether placental pathologies affect oxidative stress biomarker production, we compared their teased placenta supernatant levels taking into consideration the presence of parasites in the placenta tissue (16/120) (S1 File and Fig 3A), the accumulation of leukocytes in the intervillous space (22/120) (S1 File and Fig 3B) and the presence of malaria pigments (15/120) (S1 File and Fig 3C) (by histology). The levels of MDA (median = 1.40 vs 0.80) in the teased placenta supernatant were significantly higher in the infected than in the non-infected women (P = 0.025) (Fig 3A vs 3D; Table 3). Importantly, levels of this mediator correlated positively with increasing parasitaemia (r_s_ = 0.21, P = 0.024) (spearman rank order coefficient). On the contrary, placental supernatant levels of the oxidative stress biomarkers: NO (median = 0.06 vs 0.07) (P = 0.046), SOD (median = 54.61 vs 79.50) (P = 0.015) and to a lesser extent CAT (median = 0.90 vs 2.23) (P = 0.09) were higher in women without leukocyte accumulation in the intervillous space compared to those with leukocyte accumulation: The levels of these three mediators also correlated negatively with leukocyte accumulation in the intervillous space of the placenta [(r_s_ = -0.19, P = 0.048 for NO, r_s_ = -0.22, P = 0.016 for SOD and r_s_ = -0.16, P = 0.08 for CAT. The teased placenta supernatant levels of MDA (median = 31.4 vs 0.82) were higher in women with malaria pigments in the placenta than in those without (P = 0.021). Notably, the presence of malaria pigments correlated positively with levels of MDA (r_s_ = 0.21, P = 0.022) (Table 3). Together, these results are suggestive of the implication of oxidative stress via MDA biomarkers in the pathogenesis of placental malaria, while SOD and NO and to a lesser extent CAT may protect against PM.

**Table 3:** Median levels [95% confidence intervals] of oxidative stress markers according to placental pathologies.

Placental pathology	Oxidative Stress Marker (N = 120)				
	MDA (mM/mg)	NO (mM)	SOD (SOD unit/mg)	CAT (μM of H_2_O_2_/min/mg)	GSH (mM/mg)
Parasites in the placenta (n = 16)	1.40[1.16–1.71]	0.07[0.05–0.12]	80.39[55.5–86.1]	1.42[1.04–3.01]	55.37[52.3–66.0]
No parasites in the placenta (n = 104)	0.80[0.98–1.34]	0.07[0.07–0.08]	76.89[69.5–85.6]	2.14[2.02–3.30]	57.00[55.2–62.0]
**P**	**= 0.025**	= 0.80	= 0.70	= 0;78	= 0.70
Leukocyte accumulation (n = 22)	0.93[0.81–1.57]	0.06[0.05–0.07]	54.61[44.2–72.1]	0.90[0.86–2.30]	56.00[53.1–69.3]
No leukocyte accumulation (n = 98)	0.93[1.02–1.38]	0.07[0.07–0.08]	79.50[72.6–88.9]	2.23[2.13–3.47]	57.28[54.8–61.5]
**P**	= 0.70	**= 0.046**	**= 0.015**	**= 0.09**	= 0.59
Presence of pigments (n = 15)	1.40[1.13–1.98]	0.08[0.05–0.13]	84.67[58.4–94.1]	1.90[1.07–3.97]	60.00[55.2–74.5]
Absence of pigments (n = 105)	0.82[0.97–1.32]	0.07[0.07–0.08]	75.80[68.7–84.6]	2.66[1.96–3.21]	56.03[54.5–61.0]
**P**	**0.021**	0.37	= 0.75	0.90	0.12

Values outside the bracket correspond to the median while values in brackets correspond to the lower and upper 95% confidence intervals, using Mann-Whitney Rank Sum test. MDA: Malondialdehyde; NO: nitric oxide; SOD: Superoxide dismutase, CAT: Catalase, GSH: Glutathione. **P** = p value

### Multivariate analysis on oxidative stress biomarker production

Since there is a co-variation of parity and baby weight influencing oxidative stress biomarker production in the univariate analyses described above, and the potential effects of malaria infection, haemoglobin levels and mother age, the impact of these variables were jointly tested by multiple linear regression analysis. All the variables correlated poorly with teased placenta supernatant levels of NO. However, while baby birth weight associated with the levels of SOD (p = 0.027), GSH (P = 0.042) and to a lesser extent that of CAT (P = 0.11), haemoglobin levels associated instead with levels of MDA (P = 0.031). These results are in concordance with the univariate analysis.

## Discussion

A better understanding of the mechanisms by which placental *P*. *falciparum* infection leads to poor pregnancy outcomes is necessary for the development of diagnostic, preventive and therapeutic methods for this pathology. The sequestration of infected Red blood cells (I-RBCs) in the placenta and inflammation of the placental tissue are central to the pathophysiology of placental malaria (PM). Although oxidative stress has been reported in several placental disorders and pregnancy pathologies [8], data on PM and oxidative stress are uncommon. This study therefore sought to determine the association of *P*. *falciparum* infection and oxidative stress in placenta tissue, and whether increasing levels of oxidative stress biomarkers in the supernatants from placental tissue homogenate correlate with poor pregnancy outcomes. In general, MDA levels were significantly higher in the supernatants from the placenta tissue homogenate of women with infected placenta (impression smears). There was a similar observation in the women with regards to the presence of parasites in the placenta as well as in the group with malaria pigments in the placenta tissue (diagnosed histologically). In addition, levels of this biomarker correlated significantly and positively with the parasitaemia from placental impression smear (diagnosed microscopically), as well as with the presence of pigments in the placenta (diagnosed histologically). Levels of NO, SOD and to a lesser extent CAT associated negatively with leukocyte accumulation in that organ. These observations suggest the possible involvement of MDA in the pathogenesis of PM, while the other oxidative stress biomarkers (NO, SOD) and to a lesser extent CAT might protect against placental inflammation. Thus, *P*. *falciparum* infection might cause oxidative stress in placenta tissue. These observations corroborate previous findings where associations between peripheral plasma levels of MDA and cerebral malaria in children [14] as well as malaria infection during pregnancy in women have been reported [10]

Lipid peroxidation is the main manifestation of oxidative stress. MDA which is the end product of non enzymatic degradation of polyunsaturated fatty acids is mostly found in cell membrane. High MDA level found in the supernatant from placental tissue homogenate is indicative of increased lipid peroxidation, which might somehow damage this organ and impair foetal growth. In fact, some studies have reported that increase levels of a specific plasma muscle protein in children with cerebral malaria might lead to muscle damage and microvasculature lesions [20]. Others have demonstrated the production of free radicals (OH, H_2_O_2_), due to oxidative stress in the course of malaria infection [21, 22]. Oxidative stress is the shifting of pro-oxidant/ anti-oxidant balance usually present in normal cells towards the pro-oxidant side, which is manifested by elevated levels of free radicals responsible for cell damage. NO is a pro-oxidant marker. It can be scavenged by O_2_^-^ to form ONOO^--^, transformed to peroxy-nitrous acid, which induces the oxidation of endogenous compounds. SOD, GSH and CAT are produced in response to increasing levels of pro-oxidant markers and have antioxidant effects. A recent study reported on the decrease in the levels of SOD, and CAT, and an increase in the plasma levels of MDA with hypertension [23]. In this study, the difference in the levels of SOD, GSH, CAT and NO between malaria non infected and infected women was not significant, although the levels of MDA increased with malaria infection. This finding follows the same trend as reported elsewhere on oxidative stress and hypertension [24].

Regarding the relationship between assayed oxidative stress biomarkers and anaemia, the MDA level was higher in anaemic women. Furthermore, the level of this oxidative stress biomarker correlated negatively with the peripheral haemoglobin levels. In contrast, there was no significant association between haemoglobin and the other oxidative stress biomarkers assayed. These results might suggest oxidative stress in PM to be one of the mechanisms by which maternal anaemia may occur. This is akin to other findings showing that erythrocyte exposure to oxidative stress is associated with membrane disruption and enhanced removal from the circulation [25, 26].

The role of oxidative stress during malaria infection is still unclear. While some schools of thought suggest a protective role, others claim a relation to the physiopathology of the disease [27]. Recent studies suggest that the generation of reactive oxygen and nitrogen species (ROS and RNS) associated with oxidative stress, plays a crucial role in the development of systemic complications caused by malaria. Malaria parasite generates free radicals that might cause apoptosis in the liver. This induces changes in erythrocytes and endothelial cells, enhancing the internalization of *P*. *falciparum* parasites in tissues such as the liver and brain [28]. To the best of our knowledge, this is the first study depicting the association between MDA and the pathogenesis of PM, and suggesting the involvement of oxidative stress in the pathophysiology of placenta malaria.

Despite well documented association between poor pregnancy outcomes and placental malaria, its diagnosis during pregnancy remains a great challenge. Antenatal diagnosis of PM by Giemsa-stained blood cannot capture all the PM cases [11, 29]. This study showed a positive association between MDA levels from the supernatant of placental homogenate and PM as well as the presence of malaria pigment in the placenta tissue. In addition, a negative association was found with the maternal haemoglobin level and to a lesser extent birth weight. Therefore, the findings depict MDA as a potential biomarker for placental malaria. More so as the MDA produced in the tissue as lipid peroxide product can be detected in peripheral plasma. A positive and significant correlation was also found between the levels of SOD, CAT and birth weight, while levels of GSH and NO correlated negatively with birth weight. These observations highlight the importance of anti-oxidative biomarkers in foetal growth.

## Conclusion

Placental *P*. *falciparum* infection can cause oxidative stress of the placenta tissue with MDA being a potential biomarker of PM, which alongside GSH might lead to poor pregnancy outcomes such as anaemia and low birth weight.

## Supporting Information

S1 File Raw data set. (XLS)
